# Complete Genome Sequence of *Mycoplasma bovirhinis* Strain HAZ141_2 from Bovine Nasal Discharge in Japan

**DOI:** 10.1128/genomeA.01000-17

**Published:** 2017-09-28

**Authors:** Eiji Hata, Kazuya Nagai, Kenji Murakami

**Affiliations:** a Dairy Hygiene Unit, Division of Pathology and Pathophysiology, Hokkaido Research Station, National Institute of Animal Health (NIAH), National Agriculture and Food Research Organization (NARO), Toyohira-ku, Sapporo, Hokkaido, Japan; b Department of Veterinary Medicine, Faculty of Agriculture, Iwate University, Morioka, Iwate, Japan

## Abstract

*Mycoplasma bovirhinis*, a mycoplasmal species involved in bovine respiratory diseases, is also a commensal microorganism that inhabits the bovine respiratory and reproductive organs. We present the complete 948,039-bp genome sequence of *M. bovirhinis* strain HAZ141_2, which was isolated from bovine nasal discharge in Japan.

## GENOME ANNOUNCEMENT

*Mycoplasma bovirhinis* is a commensal microorganism that inhabits the bovine respiratory and reproductive organs and can become a secondary cause of the calf pneumonia complex at the same time (1, 2). Mycoplasmal calf pneumonia has been increasing in prevalence throughout the world along with the dissemination of large-scale livestock management and high stocking density, and the economic loss caused by this condition is considerable (3). Although the whole-genome sequences of many mycoplasma species that cause calf pneumonia have already been reported, i.e., *M. bovigenitalium* (4), *M. bovis* (5), and *M. dispar* (GenBank accession no. CP007229), that of *M. bovirhinis* has not yet been reported. Hence, we present here the whole-genome sequence of strain HAZ141_2, which was isolated in 2008 from the nasal discharge of a coughing calf in Japan.

The total genomic DNA from *M. bovirhinis* strain HAZ141_2 was prepared and subjected to 454 titanium sequencing at Iwate University, Morioka, Japan. The resulting reads were assembled *de novo* using GS De Novo Assembler software v2.7 (Roche), yielding 109 contigs with 108.5× coverage. An analysis of the contig ends together with PCR amplification and amplicon cloning showed that the 948,039-bp genome had a closed-ring structure. After performing an initial automated annotation using Microbial Genome Annotation Pipeline v2.20 at the DNA Data Bank of Japan (http://migap.ddbj.nig.ac.jp/mgap/jsp/index.jsp) (6–8), we carried out manual curation, followed by verification of potential pseudogenes by PCR and Sanger sequencing. We confirmed 629 open reading frames, 37 pseudogenes, 31 tRNAs, three sets of 16S rRNA and 23S rRNA, and two sets of 5S rRNA in this genome sequence. The guanine-cytosine content was 28.24%.

The amino acid sequences of most genes containing rRNA genes in *M. bovirhinis* HAZ141_2 exhibited high similarity to those of the genes encoded by *M. canis* (accession no. CP014281). Furthermore, a large chromosome insertion (from MBVR141_0922 to MBVR141_1042, 53.5 kb) was confirmed in this strain.

With respect to the genes of proteins involved in the production of reactive oxygen species (ROS), which have been suggested to be important mycoplasmal etiologic agents (9), the genes of proteins involved in the membrane-located ATP-binding cassette transporter system, i.e., glycerol ABC transporter ATP-binding protein (MBVR141_0395) and glycerol ABC transporter permease (MBVR141_0397, MBVR141_0665, MBVR141_0666), have been confirmed in this genome. Moreover, the genes of proteins involved in glycerol transporter (MBVR141_0783, MBVR141_0897), glycerol kinase (MBVR141_0898), and glycerol-3-phosphate dehydrogenase (MBVR141_0899) have also been confirmed in this genome, so the present strain seems to be able to produce ROS (9).

In this genome, some genes of proteins involved in the synthesis of capsular polysaccharides (galactan), which have been suggested to be important mycoplasmal etiologic agents, were absent (i.e., phosphomannomutase, UTP-glucose-1-phosphate uridyltransferase, UDP-glucose 4-epimerase, UDP-galactopyranose mutase, and BcsA glycosyltransferases), so the present strain seems not to produce galactan (10).

The genomic sequence of *M. bovirhinis* will provide a foundation for future research on this species. Ultimately, it is hoped that the present study will contribute to the reduction of mycoplasmal bovine diseases.

### Accession number(s).

This complete genome sequence has been deposited at DDBJ/EMBL/GenBank under accession no. AP018135.

## References

[1] Shimizu T, Nosaka D, Nakamura N 1973 An enzootic of calf pneumonia associated with *Mycoplasma bovirhinis*. Nihon Juigaku Zasshi 35:535–537. doi:10.1292/jvms1939.35.535. 4799971

[2] Langford EV 1975 Mycoplasma species recovered from the reproductive tracts of western Canadian cows. Can J Comp Med 39:133–138. 1125831 PMC1277433

[3] Nicholas RA, Ayling RD 2003 *Mycoplasma bovis*: disease, diagnosis, and control. Res Vet Sci 74:105–112. doi:10.1016/S0034-5288(02)00155-8. 12589733

[4] Hata E, Nagai K, Murakami K 2017 Complete genome sequence of *Mycoplasma bovigenitalium* strain HAZ 596 from a bovine vagina in Japan. Genome Announc 5(6):e01554-16. doi:10.1128/genomeA.01554-16. 28183755 PMC5331495

[5] Wise KS, Calcutt MJ, Foecking MF, Röske K, Madupu R, Methé BA 2011 Complete genome sequence of *Mycoplasma bovis* type strain PG45 (ATCC 25523). Infect Immun 79:982–983. doi:10.1128/IAI.00726-10. 21134966 PMC3028869

[6] Noguchi H, Taniguchi T, Itoh T 2008 MetaGeneAnnotator: detecting species-specific patterns of ribosomal binding site for precise gene prediction in anonymous prokaryotic and phage genomes. DNA Res 15:387–396. doi:10.1093/dnares/dsn027. 18940874 PMC2608843

[7] Lagesen K, Hallin P, Rødland EA, Staerfeldt HH, Rognes T, Ussery DW 2007 RNAmmer: consistent and rapid annotation of ribosomal RNA genes. Nucleic Acids Res 35:3100–3108. doi:10.1093/nar/gkm160. 17452365 PMC1888812

[8] Tatusov RL, Fedorova ND, Jackson JD, Jacobs AR, Kiryutin B, Koonin EV, Krylov DM, Mazumder R, Mekhedov SL, Nikolskaya AN, Rao BS, Smirnov S, Sverdlov AV, Vasudevan S, Wolf YI, Yin JJ, Natale DA 2003 The COG database: an updated version includes eukaryotes. BMC Bioinformatics 4:41. doi:10.1186/1471-2105-4-41. 12969510 PMC222959

[9] Pilo P, Frey J, Vilei EM 2007 Molecular mechanisms of pathogenicity of *Mycoplasma mycoides* subsp. *mycoides* SC. Vet J 174:513–521. doi:10.1016/j.tvjl.2006.10.016. 17157043 PMC2628566

[10] Bertin C, Pau-Roblot C, Courtois J, Manso-Silván L, Tardy F, Poumarat F, Citti C, Sirand-Pugnet P, Gaurivaud P, Thiaucourt F 2015 Highly dynamic genomic loci drive the synthesis of two types of capsular or secreted polysaccharides within the *Mycoplasma mycoides* cluster. Appl Environ Microbiol 81:676–687. doi:10.1128/AEM.02892-14. 25398856 PMC4277593

